# PTGES2 and RNASET2 identified as novel potential biomarkers and therapeutic targets for basal cell carcinoma: insights from proteome-wide mendelian randomization, colocalization, and MR-PheWAS analyses

**DOI:** 10.3389/fphar.2024.1418560

**Published:** 2024-07-05

**Authors:** Qiu-Ju Han, Yi-Pan Zhu, Jing Sun, Xin-Yu Ding, Xiuyu Wang, Qiang-Zhe Zhang

**Affiliations:** ^1^ State Key Laboratory of Medicinal Chemical Biology and College of Pharmacy, Tianjin Key Laboratory of Molecular Drug Research, Nankai University, and the Haihe Laboratory of Cell Ecosystem, Tianjin, China; ^2^ Department of Neurosurgery, Tianjin First Central Hospital, School of Medicine, Nankai University, Tianjin, China

**Keywords:** biomarkers, basal cell carcinoma, proteome-wide mendelian randomization, colocalization analysis, MR-pheWAS, drug targets, plasma proteins, single nucleotide polymorphisms

## Abstract

**Introduction:**

Basal cell carcinoma (BCC) is the most common skin cancer, lacking reliable biomarkers or therapeutic targets for effective treatment. Genome-wide association studies (GWAS) can aid in identifying drug targets, repurposing existing drugs, predicting clinical trial side effects, and reclassifying patients in clinical utility. Hence, the present study investigates the association between plasma proteins and skin cancer to identify effective biomarkers and therapeutic targets for BCC.

**Methods:**

Proteome-wide mendelian randomization was performed using inverse-variance-weight and Wald Ratio methods, leveraging 1 Mb cis protein quantitative trait loci (cis-pQTLs) in the UK Biobank Pharma Proteomics Project (UKB-PPP) and the deCODE Health Study, to determine the causal relationship between plasma proteins and skin cancer and its subtypes in the FinnGen R10 study and the SAIGE database of Lee lab. Significant association with skin cancer and its subtypes was defined as a false discovery rate (FDR) < 0.05. pQTL to GWAS colocalization analysis was executed using a Bayesian model to evaluate five exclusive hypotheses. Strong colocalization evidence was defined as a posterior probability for shared causal variants (PP.H4) of ≥0.85. Mendelian randomization-Phenome-wide association studies (MR-PheWAS) were used to evaluate potential biomarkers and therapeutic targets for skin cancer and its subtypes within a phenome-wide human disease category.

**Results:**

PTGES2, RNASET2, SF3B4, STX8, ENO2, and HS3ST3B1 (besides RNASET2, five other plasma proteins were previously unknown in expression quantitative trait loci (eQTL) and methylation quantitative trait loci (mQTL)) were significantly associated with BCC after FDR correction in the UKB-PPP and deCODE studies. Reverse MR showed no association between BCC and these proteins. PTGES2 and RNASET2 exhibited strong evidence of colocalization with BCC based on a posterior probability PP.H4 >0.92. Furthermore, MR-PheWAS analysis showed that BCC was the most significant phenotype associated with PTGES2 and RNASET2 among 2,408 phenotypes in the FinnGen R10 study. Therefore, PTGES2 and RNASET2 are highlighted as effective biomarkers and therapeutic targets for BCC within the phenome-wide human disease category.

**Conclusion:**

The study identifies PTGES2 and RNASET2 plasma proteins as novel, reliable biomarkers and therapeutic targets for BCC, suggesting more effective clinical application strategies for patients.

## Introduction

The skin, our largest organ, is directly exposed to various external factors, leading to the emergence of skin cancer among humans (Byrd et al., 2018). In recent years, skin cancer has become one of the most prevalent malignancies (Notarstefano et al., 2019; Ashraf et al., 2020; Siegel et al., 2023), globally representing the majority of malignant tumors (Perez et al., 2022). Surveys show a consistent rise in its incidence compared to other cancers (Kong et al., 2018), categorizing it broadly into melanoma and non-melanoma skin cancer (NMSC) (Balasubramanian et al., 2019). Melanoma, a rare and highly dangerous variant, accounts for only 1.7% of skin cancers, according to statistics from the American Cancer Society (Saginala et al., 2021). NMSC includes Merkel cell carcinoma, adnexal carcinoma, and dermatofibrosarcoma protuberans but predominantly comprises basal cell carcinoma (BCC) and squamous cell carcinoma (SCC), collectively known as keratinocyte carcinomas (Lomas et al., 2012; Nehal and Bichakjian, 2018). BCC represents over 80% of NMSC cases (Rubin et al., 2005). Current treatments for BCC include surgical intervention with radiotherapy, cryotherapy, and photodynamic therapy (Kuflik and Gage, 1991; Silverman et al., 1992; Wong et al., 2003). However, there are no established biomarkers or therapeutic targets for guiding effective treatment.

The development of BCC is influenced by various factors, including levels of hair and skin pigmentation, sun exposure, immunosuppression, and exposure to harmful chemicals or ionizing radiation (Situm et al., 2008; Collins et al., 2019; Kim et al., 2019). Genetic variants in pigmentation genes (RALY, IRF4, MC1R, OCA2, SLC45A2, and TYR), as well as immune genes (HLA and LPP), have been implicated in the susceptibility to BBC development (Chahal et al., 2016). Prolonged exposure to ultraviolet radiation leads to the secretion of inflammatory cytokine (TNF-α, IL-1β, IL-6, IL-10) that cause erythema, photoaging, immunosuppression, and DNA damage, ultimately contributing to BCC formation (Wong et al., 2003; Matsumura and Ananthaswamy, 2004; Ikehata and Ono, 2011). Survivors of nuclear disasters, radiation-exposed workers, and patients undergoing long-term radiation therapy exhibit a higher incidence rate of BCC compared to other malignant neoplasms. DNA damage and Shh signaling are identified as crucial mechanisms underlying the development of radiation-induced BCC (Li and Athar, 2016). These facts highlight that genetic variations and protein interactions play a crucial role in the pathogenesis of BCC serving as potential diagnostic markers or therapeutic targets for this disease.

Genome-wide association studies (GWAS) aim to identify genetic variants associated with disease outcomes or traits by analyzing entire genomes of large populations (Loos, 2020; Uffelmann et al., 2021). Variants influencing gene expression are called expression quantitative trait loci (eQTLs), while those affecting DNA methylation are known as methylation quantitative trait loci (mQTLs) (Lin et al., 2020). GWAS has uncovered over 70,000 associations (Buniello et al., 2019), aiding in drug target identification (Schmidt et al., 2020), drug repurposing (Reay and Cairns, 2021), predicting clinical trial side effects (Nguyen et al., 2019), and patient reclassification (Christiansen et al., 2021). The human proteome offers potential therapeutic targets, with the plasma proteome containing proteins actively secreted, shed, or leaked into circulation for executing their functions or inter-tissue communication (Gudjonsson et al., 2022). Integrating human genetics with population-scale proteomics can bridge the gap between the genome and diseases, providing insights into health and the impact of lifestyle and environment on disease (Sun et al., 2023). The associations between protein levels and disease are often insufficient to distinguish causality. However, combining protein quantitative trait loci (pQTLs) and disease variants’ associations and colocalization can differentiate cause and effect, elucidate pathogenesis, and identify drug targets (Eldjarn et al., 2023).

Large-scale GWASs have been conducted to understand the genetic basis of skin cancers, revealing over 140 eQTLs and mQTLs (Flint, 2013; Adolphe et al., 2021; Seviiri et al., 2022). However, potential biomarkers and therapeutic targets from plasma proteins (pQTLs) in BBC remain unclear. By leveraging overlapping genetics, multivariate GWAS approaches, including efficient colocalization algorithms, can identify novel risk regions (GWAS to the people, 2018; Turley et al., 2018). Recently, an agnostic strategy integrating phenome-wide association study (PheWAS) and Mendelian randomization (MR) (termed MR-PheWAS) has been proposed to establish causal relationships with previously unconsidered traits. In this study, to deepen our understanding of potential biomarkers and therapeutic targets of BCC, we used MR-PheWAS based on inverse-variance-weight (IVW) and Wald Ratio (Kuflik and Gage, 1991) to minimize reverse bias and residual confounding bias, as it does not affect results through pathways other than exposure (GWAS to the people, 2018; Turley et al., 2018; Eldjarn et al., 2023). Ultimately, PTGES2 and RNASET2 were identified as potential biomarkers and therapeutic targets for BCC through integrating proteome-wide MR, colocalization, and MR-PheWAS analyses.

## Materials and methods

### Experimental process

**FIGURE 1:**
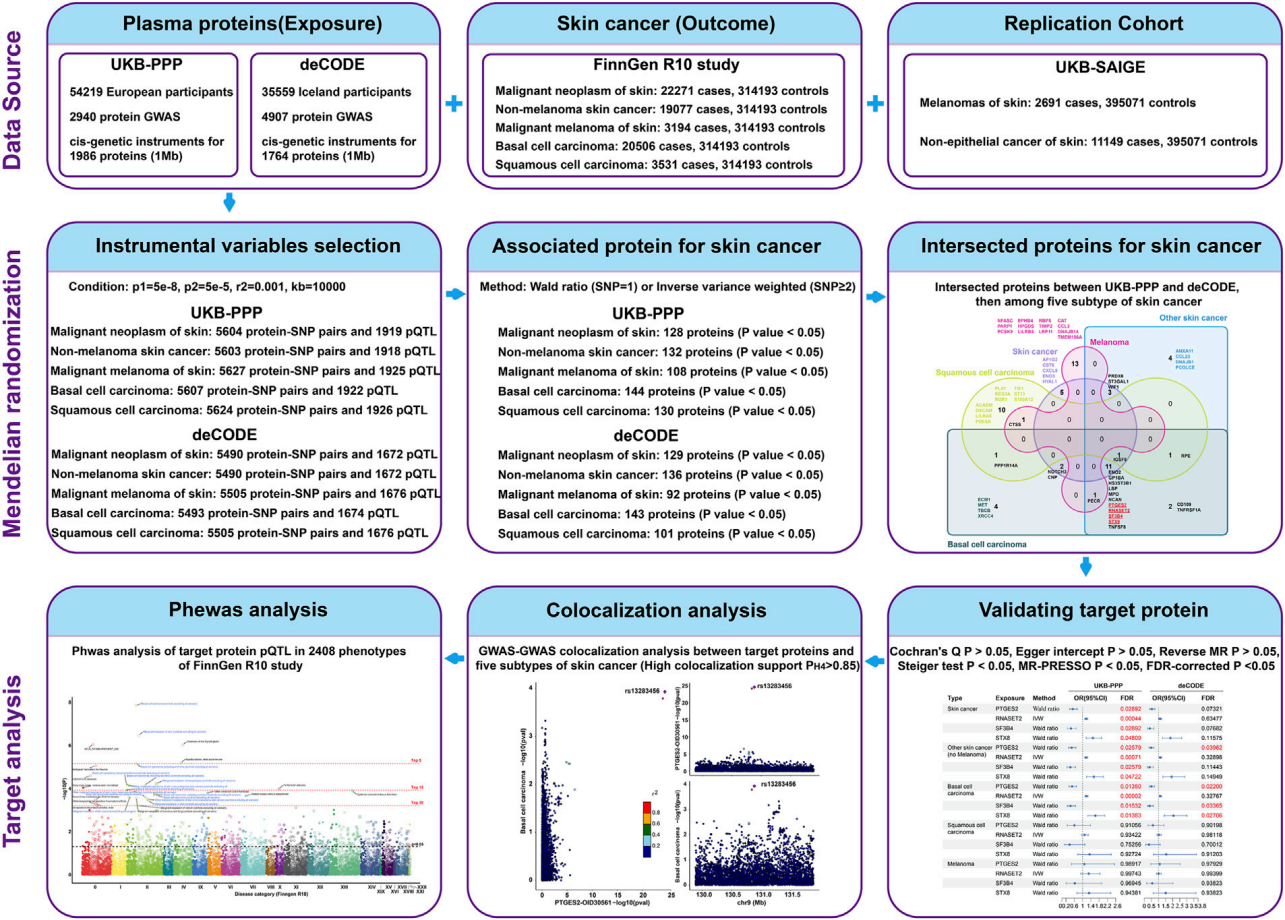
Overview of the study design.

The study design overview is shown in Figure 1, with all analyses based on summary-level data detailed in Supplementary Table S1. Initially, we analyzed the associations of genetically predicted plasma proteins in the UK Biobank Pharma Proteomics Project (UKB-PPP) and deCODE studies with skin cancer in the FinnGen R10 study. Subsequently, we investigated the associations of these proteins with four subtypes of skin cancer (NMSC, BCC, SCC, and melanoma) to understand their roles in disease development. To confirm a causal relationship, colocalization analyses were performed using GWAS-to-GWAS comparisons. Furthermore, the efficacy of skin cancer-associated proteins was assessed across various human diseases through MR-PheWAS on 2,408 phenotypes from the FinnGen R10 study dataset. Finally, we validated key findings within specific subtypes of skin cancer using data from the UK Biobank database.

### Data sources of plasma proteins

We utilized data from two large-scale GWAS studies on plasma protein levels, including the UK Biobank Pharma Proteomics Project (UKB-PPP) and the deCODE Health Study. The UKB-PPP provided 2,940 protein GWAS from 54,219 participants (Sun et al., 2023), which were measured with the antibody-based Olink Explore 3072 platform. While deCODE generated 4,907 protein GWAS from 35,559 Icelanders (Ferkingstad et al., 2021a), measured by the SomaScan v4 platform. Instrumental variables were selected as 1 Mb cis-SNPs of plasma proteins with *p* < 5 × 10^−8^, with linkage disequilibrium estimated based on the 1000 Genomes European panel. For the two-sample MR analysis, we obtained cis-genetic instruments for 1,986 and 1,764 proteins from the UKB-PPP and deCODE studies, respectively. Overlapping proteins with genetic instruments from both studies were examined to ensure consistency across different proteomic profiling platforms.

### Data sources of skin cancer and its subtypes

Data on plasma protein-associated SNPs with skin cancer and its subtypes were obtained from the FinnGen study and UKB database. The FinnGen R10 study provided the latest release data on skin cancer and its subtypes, including cases of skin cancer (22,271), NMSC (19,077), BCC (20,506), SCC (3,531), and melanoma of the skin (3,194), accompanied by identical controls, totaling 314,193 individuals. Replication datasets for skin cancer included melanomas (2,691 cases, 395,071 controls) and non-epithelial skin cancer (11,149 cases, 395,071 controls) from the Lee lab in the UKB database. As both the replication data for skin cancer and plasma proteins of UKB-PPP belong to the UKB database, there may be sample overlap between them, with the protein sample size ranging from 9,216 to 34,090 and the replication data size ranging from 39,7762 to 40,6220. Based on these data, the max repetitive rate was 8.6%, with the validity exceeding 90%, to ensure effective MR (Pierce and Burgess, 2013; Burgess et al., 2016). However, no sample overlaps were observed between other plasma proteins and skin cancer datasets in this study. For the reverse MR analysis, genetic variants associated with skin cancer and its subtypes at *p* < 5 × 10^−8^ and with low linkage disequilibrium (*R* ^2^ < 0.001) were selected as instrument variables for skin cancer.

### Mendelian randomization analysis

After excluding SNPs with *p* < 5 × 10^5^ and plasma proteins lacking genetic instrumental variables in the skin cancer data, we conducted a batch analysis of two-sample MR. The strength of the instrumental variables was assessed using the F statistic. Associations between plasma proteins and the studied outcomes were estimated using the Wald ratio and IVW fixed-effect model or IVW multiplicative random effects (Burgess et al., 2015), along with their corresponding odds ratios (ORs) and confidence intervals (CIs). To correct for multiple testing in skin cancer analysis, the false discovery rate (FDR) method with a significance threshold of <0.05 was applied (Glickman et al., 2014). In addition, reverse MR analysis estimated the associations of skin cancer liability with identified protein levels to explore potential reverse causation. MR analyses were performed using TwoSampleMR and MendelianRandomization packages in R software (4.3.1). Causal directionality was ensured through Steiger tests at a significance level of *p* < 0.05, heterogeneity was checked using Cochran’s Q test with a criterion of *p* > 0.05 (Burgess et al., 2013), and pleiotropy was evaluated using Egger intercept test with a criterion of *p* > 0.05 (Bowden et al., 2015). Outlier SNPs were identified using MR-Presso based on *p* > 0.05 as a criterion (Verbanck et al., 2018).

### Colocalization analysis

A colocalization analysis was conducted to assess whether the associations between proteins and skin cancer, including its subtypes, were influenced by linkage disequilibrium. This analysis utilized a Bayesian model that considered five exclusive hypotheses: 1) no association with either trait; 2) association with trait 1 alone; 3) association with trait 2 alone; 4) association with both traits, but distinct causal variants exist for each trait; and 5) association with both traits, sharing the same causal variant (Yuan et al., 2023). Each hypothesis (H0, H1, H2, H3, and H4) was assigned a posterior probability. We set the prior probabilities for the SNP being associated with trait 1 alone at 1 × 10^−4^, for the SNP being associated with trait 2 alone at 1 × 10^−4^, and for the SNP being associated with both traits at 1 × 10^−5^. Strong colocalization was defined as a posterior probability for shared causal variants (PP.H4) ≥ 0.85, and medium colocalization indication was indicated by 0.5 < PP.H4 < 0.85. The coloc package in R software (version 4.3.1) was used for this analysis.

### Mendelian randomization-phenome-wide association studies (MR-PheWAS)

The FinnGen provided genetic association results for 2,405 binary endpoints and 3 quantitative endpoints (HEIGHT_IRN, WEIGHT_IRN, and BMI_IRN) from freeze 10 (December 2023) (Kurki et al., 2023), involving 41,2181 individuals. Skin cancer-associated SNPs were selected as instrumental variables for batch MR analysis with these 2,408 phenotypes in the FinnGen R10 study. MR-PheWAS utilized the Wald ratio and IVW methods, with parameters and criteria consistent with those in the initial MR analysis. Newly identified phenotypes associated with skin cancer-associated proteins underwent colocalization analysis to verify causal relationships.

## Results

### Associations between plasma proteins and skin cancer

**FIGURE 2:**
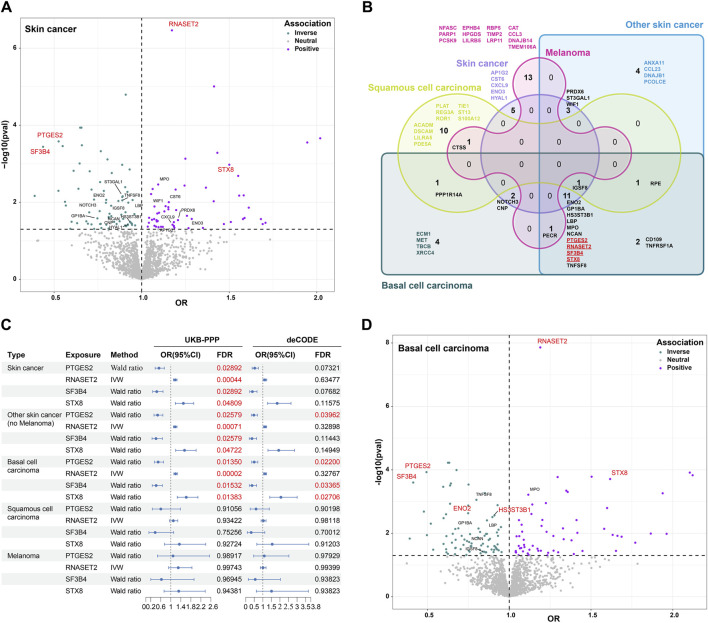
Association between plasma proteins and skin cancer and its subtypes using MR analysis. **(A)**. Volcano plot displaying the association of proteins with skin cancer based on odds ratio (OR) in MR analysis. Twenty-two intersected proteins significantly associated with skin cancer in the UKB-PPP and deCODE studies (*p* < 0.05). Blue denotes a protective effect, purple indicates a risk factor, and gray represents neutrality. **(B)**. Venn diagram illustrating the proteins associated with skin cancer and its subtypes through MR analysis (*p* < 0.05). **(C)**. Odds ratios (ORs), 95% confidence intervals (CIs), and false discovery rate (FDR) for the effect of plasma proteins on skin cancer and its subtypes estimated using the IVW or Wald ratio approaches of MR. **(D)**. Volcano plot showing the association of proteins with BCC based on odds ratio (OR) in MR analysis (*p* < 0.05). Twelve intersected proteins in skin cancer, non-melanoma skin cancer, and BCC were highlighted. The red fonts represent significantly associated proteins (FDR <0.05).

To discover potential biomarkers or therapeutic targets for skin cancers, we first investigated the causal relationship between plasma proteins and total skin cancers. After excluding plasma proteins without genetic instruments in skin cancer data, MR analysis included 1,919 proteins in the UKB-PPP and skin cancer, as well as 1,672 proteins in the deCODE and skin cancer in the FinnGen R10 study, with an intersection of 837 proteins in subsequent analyses. These findings obtained from analyzing plasma proteins and skin cancer are presented in Figure 2. In the intersected analysis of two plasma proteins data, genetically predicted levels of 22 proteins were significantly associated with skin cancer risk (*p* < 0.05, Figure 2A). Notably, these associations were consistently observed in both the UKB-PPP and deCODE studies for all identified proteins (Supplementary Tables S2, S3). Per standard deviation increase in genetically predicted levels of protein, the OR of skin cancer ranged from 0.44 (95% CI = 0.28–0.69) for splicing factor 3B subunit 4 (SF3B4) to 1.50 (95% CI = 1.18–1.91) for Syntaxin-8 (STX8) (Supplementary Figure S1; Supplementary Table S2). Furthermore, prostaglandin E synthase 2 (PTGES2), ribonuclease T2 (RNASET2), SF3B4, and STX8 were significantly associated with skin cancer risk after FDR correction for multiple testing (FDR <0.05, Supplementary Figure S1). Genetically predicted levels of the other studied proteins were not associated with skin cancer risk (Supplementary Tables S2, S3).

### Associations between plasma proteins and the subtypes of skin cancer

To further understand the role of plasma proteins in specific skin cancers, we investigated the causal relationship between these proteins and NMSC, BCC, SCC, and melanoma. We identified 23 intersected proteins with a *p*-value <0.05 in both the UKB-PPP and deCODE studies for NMSC (predominantly including BCC and SCC), along with 23, 14, and 15 intersected proteins for BCC, SCC, and melanoma of the skin, respectively (Figure 2B; Supplementary Table S4). However, FDR correction for multiple testing revealed no significant associations for SCC and melanoma of the skin (Supplementary Table S5). Interestingly, PTGES2, RNASET2, SF3B4, and STX8, associated with skin cancer risk, were also significantly associated with NMSC and BCC risks (Figures 2B,C). These findings suggest that these four proteins are specifically associated with BCC but not with SCC and melanoma of the skin. PTGES2 and SF3B4 exhibited a protective effect against BCC (OR < 1), while RNASET2 and STX8 were identified as risk factors for BCC (OR < 1) in both the UKB-PPP and deCODE studies. Furthermore, FDR-corrected *p*-value <0.05 was observed for gamma-enolase (ENO2) and heparan sulfate glucosamine 3-O-sulfotransferase 3B1 (HS3ST3B1) with BCC, with OR values <1 indicating a protective effect against BCC (Figure 2D; Supplementary Table S6). In addition, reverse MR results indicated that genetic liability to skin cancer and its subtypes were not associated with levels of six blood proteins, including PTGES2, RNASET2, SF3B4, STX8, ENO2, and HS3ST3B1, after FDR correction. These associations were consistent in a series of analyses (Supplementary Table S7). Collectively, these results demonstrate that PTGES2, RNASET2, SF3B4, STX8, ENO2, and HS3ST3B1 are significantly associated with BCC.

### PTGES2 and RNASET2 as potential biomarkers and therapeutic targets of BCC

**FIGURE 3:**
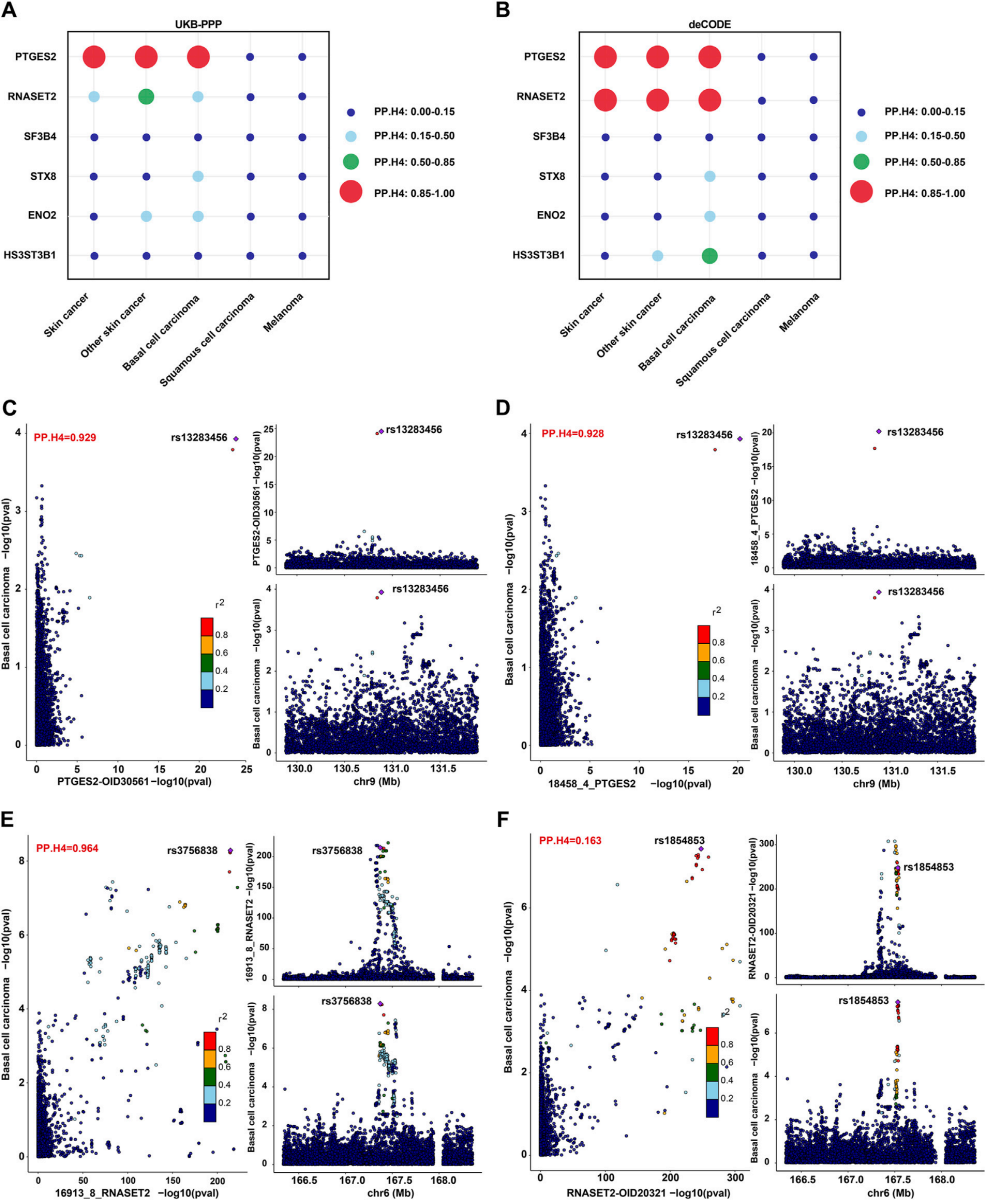
Colocalization analysis on the associations between plasma proteins and skin cancer and its subtypes. **(A,B)**. PP.H4 values of colocalization analysis for PTGES2, RNASET2, SF3B4, STX8, ENO2, and HS3ST3B1 in the UKB-PPP **(A)** or deCODE **(B)** studies with skin cancer and its subtypes. **(C,D)**. LocusCompare plot for colocalization of pQTL (PTGES2) and BCC susceptibility in the UKB-PPP **(C)** and deCODE **(D)** studies. **(E,F)**. LocusCompare plot for colocalization of pQTL (RNASET2) and BCC susceptibility in the deCODE **(E)** and UKB-PPP **(F)** studies.

To further determine the associations between plasma proteins and skin cancer and its subtypes, a colocalization analysis of GWAS-to-GWAS was conducted. In our study, strong colocalization was defined as a posterior probability (PP.H4) value ≥ 0.85. Among the six proteins identified through MR analysis about BCC, PTGES2 and RNASET2 showed strong evidence of colocalization (PP.H4 ≥ 0.85), which was also observed for skin cancer and NMSC (Figures 3A,B; Supplementary Tables S8, S9). HS3ST3B1 exhibited moderate support for colocalization in the deCODE study (0.85 > PP.H4 ≥ 0.5) (Figure 3B; Supplementary Table S9). However, SF3B4, STX8, and ENO2 did not show significant colocalization (PP.H4 < 0.5) (Figures 3A,B; Supplementary Tables S8, S9). LocusCompare plots further indicated that the BCC GWAS and PTGES2 or RNASET2 pQTL associations probably represent a true colocalization event (Figures 3C–F).

### Verification analysis of BCC liability with levels of six proteins

To validate our findings, we conducted two-sample MR, colocalization, and reverse MR analyses involving six proteins associated with skin cancer subtypes according to the ICD10 classification in the UKB database of Lee lab. The two sample MR results revealed that RNASET2, SF3B4, and HS3ST3B1 were significantly associated with non-epithelial skin cancer (Supplementary Table S10). Colocalization results found that RNASET2 was highly colocalized with non-epithelial skin cancer in both the UKB-PPP (PP.H4 = 0.973) and deCODE (PP.H4 = 0.939) studies but not with melanoma (Supplementary Figure S2; Supplementary Tables S11, S12). However, PTGES2, SF3B4, STX8, ENO2, and HS3ST3B1 did not show any significant colocalization (PP.H4 < 0.5) (Supplementary Tables S11, S12). Reverse MR results indicated that the skin cancer subtypes were not associated with levels of six plasma proteins after FDR correction (FDR >0.05) (Supplementary Table S7).

### PTGES2 and RNASET2 as effective biomarkers and therapeutic targets for BCC in phenome-wide

**FIGURE 4:**
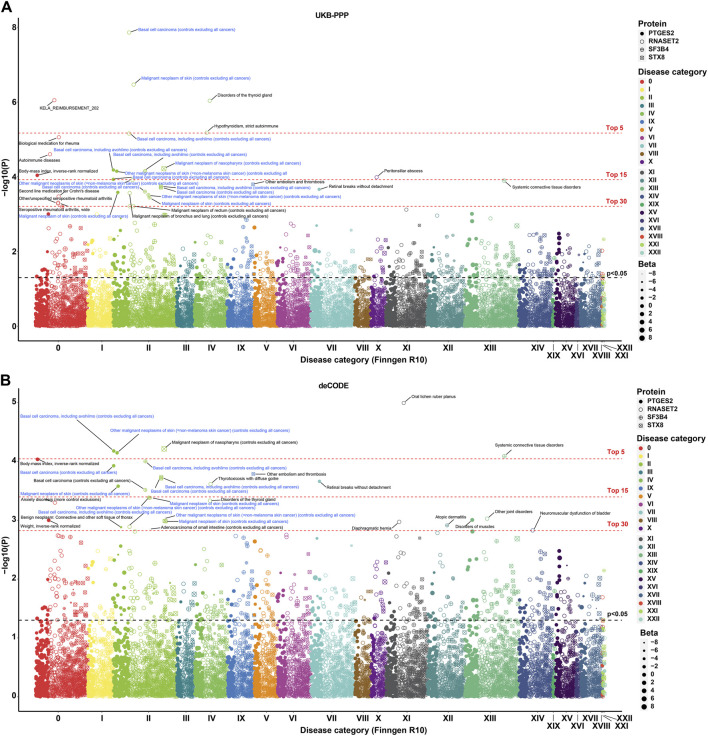
MR-PheWAS analysis for identified biomarkers and therapeutic targets. **(A)**. MR-PheWAS analysis between PTGES2, RNASET2, SF3B4, and STX8 in the UKB-PPP study and 2,408 phenotypes in the FinnGen R10 study. **(B)**. MR-PheWAS analysis between PTGES2, RNASET2, SF3B4, and STX8 in the deCODE study and 2,408 phenotypes in the FinnGen R10 study.

To determine the efficacy of PTGES2 and RNASET2 for BCC across a broad spectrum of human diseases, we conducted MR-PheWAS analysis on 2,408 phenotypes from the FinnGen R10 study. Among the top 30 phenotypes shown in Figure 4, the primary diseases were BCC or included BCC phenotypes rather than SCC and melanoma. BCC, malignant neoplasms of the skin, and gastrointestinal endpoints (KELA_REIMBURSEMENT_202) emerged as the three most significant phenotypes out of 9,621 protein-phenotype pairs, which were significantly associated with RNASET2 in the UKB-PPP study (Figure 4A; Supplementary Table S13). Similarly, BCC, malignant neoplasms of skin, and body-mass index (BMI, a quantitative endpoint) showed significant associations with PTGES2 as the top three most significant phenotypes in the UKB-PPP (9,621 protein-phenotype pairs) and deCODE (9,620 protein-phenotype pairs) studies (Figures 4A,B; Supplementary Tables S13, S14). Colocalization analysis revealed no colocalization relationship between quantitative endpoint BMI or gastrointestinal endpoints and either RNASET2 or PTGES2 (Supplementary Figures S3A,B; Supplementary Table S15). Overall, these results demonstrate that PTGES2 and RNASET2 serve as effective biomarkers and therapeutic targets for BCC within the phenome-wide human disease category.

Given the high PH3 values of SF3B4 and STX8 with some phenotypes associated with BCC (PH3 > 0.975), we further analyzed these phenome-wide maps to explore possible causality. For SF3B4, the top three most significant phenotypes were BCC, including outpatient registration cases, systemic connective tissue disorders, and BCC, excluding outpatient registration cases. Meanwhile, for STX8, the most significant phenotypes were malignant neoplasm of the nasopharynx, other embolism and thrombosis, and BCC, including outpatient registration cases in both the UKB-PPP and deCODE studies (Figures 4A,B; Supplementary Tables S13, S14). Furthermore, STX8 was colocalized with other embolisms and thrombosis (PP.H4 > 0.80), suggesting a possible mechanism for the association between STX8 and BCC (Supplementary Figure S4; Supplementary Table S16).

## Discussion

Molecular quantitative trait locus mapping is crucial for understanding the functional implications of human genetic variants (Aguet et al., 2023). pQTL analysis provides insights into the impact of systemic genetic variations on physiological and disease states, aiding biomarker discovery and disease relevance studies (Ferkingstad et al., 2021b). Integrating pQTL analysis with cancer research enhances strategies for diagnosis, prevention, and treatment across various diseases (Suhre et al., 2021). In this study, we investigated associations between plasma proteins and skin cancer using 1,986 cis-pQTLs of UKB-PPP and 1,764 cis-pQTLs of deCODE. PTGES2, RNASET2, SF3B4, and STX8 showed significant associations with skin cancer after FDR correction. Further MR analysis revealed that these proteins were specifically associated with NMSC (predominantly including BCC and SCC), but not melanoma. To identify associations with BCC and SCC specifically, we conducted MR analysis, finding PTGES2, RNASET2, SF3B4, STX8, ENO2, and HS3ST3B1 significantly associated with BCC at FDR <0.05. Among these six proteins, only RNASET2 had been previously reported as a BCC susceptibility gene in eQTL studies (Chahal et al., 2016; Liyanage et al., 2019; Seviiri et al., 2022). Bayesian colocalization analysis demonstrated that PTGES2 and RNASET2 can collocate with BCC. Furthermore, MR-PheWAS highlighted PTGES2 and RNASET2 as effective biomarkers and therapeutic targets for BCC.

Our study identified PTGES2 as a robust protective factor for BCC, with OR of 0.497 (deCODE) and 0.494 (UKB-PPP), demonstrating significant association and colocalization with BCC in both studies. PTGES2, also known as microsomal prostaglandin E synthase 2, is a crucial terminal enzyme in the synthesis pathway of prostaglandin E2 (PGE2) (Tsuge et al., 2019). It converts cyclooxygenase (COX)-synthesized prostaglandin H2 to PGE2 (Kudo and Murakami, 2005; Park et al., 2006; Chaudhry et al., 2010), regulating inflammation, immune responses, and tumor development (Kalinski, 2012; Karpisheh et al., 2019; Finetti et al., 2020). Tumor-derived PGE2 can induce dysfunction in intratumoral cDC1s, impairing their local capacity to coordinate anti-cancer CD8^+^ T cell responses (Bayerl et al., 2023). PGE2 also promotes Th1 differentiation and Th17 cell expansion, exacerbating immune-related inflammation (Yao et al., 2009). While blocking PGE2 signaling for cancer-associated fibroblasts inhibits breast cancer growth but promotes metastasis (Elwakeel et al., 2022). Despite this, the effects of PTGES2 on BCC have not been previously investigated. Previous studies have shown that COX-2 contributes to NMSC development in mouse skin models (Rundhaug et al., 2011). Interestingly, our research found that plasma proteins containing PTGES2 were favorable for NMSC development, with OR of 0.476 (deCODE) and 0.472 (UKB-PPP). The population of NMSC mainly consisted of BCC and SCC; however, we did not observe an association between PTGES2 and SCC in our study. These results suggest distinct functional roles for PTGES2 in plasma proteins during BCC development.

RNASET2 belongs to the Rh/T2/S-glycoprotein family and possesses ribonuclease function (Trubia et al., 1997). It is localized on chromosome 6q27, a region associated with human malignancies and chromosomal rearrangement (Liu et al., 2023). RNASET2 has been implicated in various pathophysiological processes, exhibiting pleiotropic roles. In clear cell renal cell carcinoma, high RNASET2 expression is linked to poor prognosis, with its silencing inhibiting the migration capacity and pro-angiogenesis of renal cancer cells (Liu et al., 2023). Conversely, in ovarian cancer, RNASET2 is a tumor suppressor by recruiting M1 macrophages to tumoral tissues (Ji et al., 2021; Bruno et al., 2022). RNASET2 also is vital for ROS propagation during oxidative stress-induced cell death (Caputa et al., 2016). Its expression could be induced by hydrogen peroxide, UV irradiation, and inflammatory triggers. To curb RNASET2 overexpression, avoiding oxidative stressors like UV radiation and supplementing with antioxidants are effective strategies. Our findings indicate that RNASET2 colocalizes with BCC and may serve as a novel risk factor for this disease. However, no direct relationship between RNASET2 and BCC has been previously established. Interestingly, a GWAS study conducted on the Chinese Han population suggested a hazardous association between vitiligo and RNASET2 (Quan et al., 2010). This suggests that understanding the role of RNASET2 could offer valuable insights into the pathogenesis of BCC and potentially contribute to the development of therapeutic strategies.

The pQTL-to-GWAS colocalization analysis is a valuable tool for determining whether proteins and phenotypes share the same causal variation locus in the same region, thereby strengthening the evidence supporting their association (Pietzner et al., 2021). In our study, PTGES2 showed robust colocalization with BCC, supported by a high posterior probability in both the UKB-PPP and deCODE studies. Conversely, RNASET2 exhibited strong colocalization with BCC only in the deCODE study, with moderate support for colocalization with NMSC in the UKB-PPP study. SNP rs13283456 and SNP rs3756838 were identified as top SNPs for PTGES2 and RNASET2, respectively. This novel finding has not been previously reported and may stem from differences in detection methods, population structures, and genetic backgrounds between the two cohorts. Notably, SNP rs13283456 is a coding non-synonymous variant within PTGES2 that has shown significant association with primary graft dysfunction in a previous study (Diamond et al., 2014). In the replication test of ICD10 classified skin cancer subtypes, SNPs rs11575078 and rs2247315 emerged as top SNPs for analyzing colocalizaton between RNASET2 and non-epithelial skin cancer types in both UKB-PPP and deCODE studies. These analyses contribute to uncovering potential disease mechanisms and advancing personalized medicine (van der Wijst et al., 2018).

MR-PheWAS plays a crucial role in establishing causal links between genes and the phenome, aiding in the assessment of potential pleiotropy and side effects of potential drug targets (Cao et al., 2023). In our study, which analyzed 2,408 phenotypes in the FinnGen R10 study (Francis et al., 2022), BCC emerged as the most significant phenotype associated with PTGES2 and RNASET2, affirming their reliability as biomarkers and therapeutic targets for BCC. In addition, BMI and gastrointestinal endpoints were among the top three most significant phenotypes linked to PTGES2 and RNASET2. Despite these associations, colocalization analysis revealed no evidence of a colocalization relationship between BMI and gastrointestinal endpoints with either RNASET2 or PTGES2. This suggests that potential side effects resulting from drugs targeting these genes are likely to be minimal. These findings from MR-PheWAS provide crucial insights for the development of more effective treatment strategies in disease research and clinical practice.

The study has five limitations. First, it may be subject to potential bias due to focusing on a single population of European descent. Including individuals from diverse ethnic backgrounds could enhance the generalizability of the research findings. Second, a similar alternative data was chosen due to the unavailability of suitable BCC datasets in the replication study, which may affect the reliability of the study results. Future studies should aim for more appropriate dataset validation. Third, heterogeneity might be introduced using two different protein analyzing platforms, even though an intersection protein approach was adopted to mitigate this issue. Fourth, the study used cis-pQTLs to investigate the causal relationship, potentially overlooking the impact of other regulatory elements and environmental factors on the disease. Incorporating trans-pQTLs could provide a comprehensive understanding of the disease. Lastly, despite implementing rigorous measures to mitigate bias, the MR, colocalization, and PheWas analyses remain susceptible to unmeasured confounding or pleiotropy, which could bias the findings. Future studies should aim to integrate multi-omics data to gain a comprehensive understanding of BCC pathogenesis. Furthermore, future investigations should aim to elucidate the *in vitro* and *in vivo* functions of RNASET2 or PTGES2, as these associations were established through rigorous *in silico* analyses.

## Conclusion

In our study, we identified six plasma proteins, including PTGES2, RNASET2, SF3B4, STX8, ENO2, and HS3ST3B1 (previously unknown in eQTL and mQTL, except for RNASET2), that were significantly associated with BCC after FDR correction in the UKB-PPP and deCODE studies (FDR<0.05). Among these proteins, PTGES2 showed a protective effect against BCC (OR<1), while RNASET2 was identified as a risk factor for BCC (OR<1). Furthermore, colocalization analysis provided strong evidence of PTGES2 and RNASET2 being associated with BCC (PP.H4 ≥ 0.85). MR-PheWAS analysis confirmed that PTGES2 and RNASET2 were significantly associated with BCC in phenome-wide. These findings highlight the potential of PTGES2 and RNASET2 as reliable biomarkers and therapeutic targets for BCC within the phenome-wide human disease category.

## Data Availability

The original contributions presented in the study are included in the article/Supplementary Material, further inquiries can be directed to the corresponding authors.
