# Supplementary material for: PTGES2 and RNASET2 identified as novel potential biomarkers and therapeutic targets for basal cell carcinoma: insights from proteome-wide mendelian randomization, colocalization, and MR-PheWAS analyses
Source: Front Pharmacol. 2024 Jul 5;15:1418560. doi: 10.3389/fphar.2024.1418560 (PMC11257982; doi:10.3389/fphar.2024.1418560)
Supplement: Supplementary file 2 [file DataSheet2.docx]

**PTGES2 and RNASET2 identified as novel potential biomarkers and therapeutic targets for basal cell carcinoma****: Insights from proteome-wide mendelian randomization, colocalization, and** **MR-PheWAS analyses**

Qiu-Ju Han^1#^, Yi-Pan Zhu^1#^, Jing Sun^1^, Xin-Yu Ding^1^, Xiuyu Wang^2^*, and Qiang-Zhe Zhang^1^*

*^1^ National Key Laboratory of Medicinal Chemical Biology and College of Pharmacy, Tianjin Key Laboratory of Molecular Drug Research, Nankai University, and the Haihe Laboratory of Cell Ecosystem, Tianjin 300350, China*

*^2^ Department of Neurosurgery, Tianjin First Central Hospital, School of Medicine, Nankai University, Tianjin, 300192, China.*

^#^ Qiu-Ju Han and Yi-Pan Zhu contributed equally to this work

* Correspondence: [zhangqiangzhe@nankai.edu.cn](mailto:zhangqiangzhe@nankai.edu.cn); wangkaiyu911@126.com

**Supplementary Information**


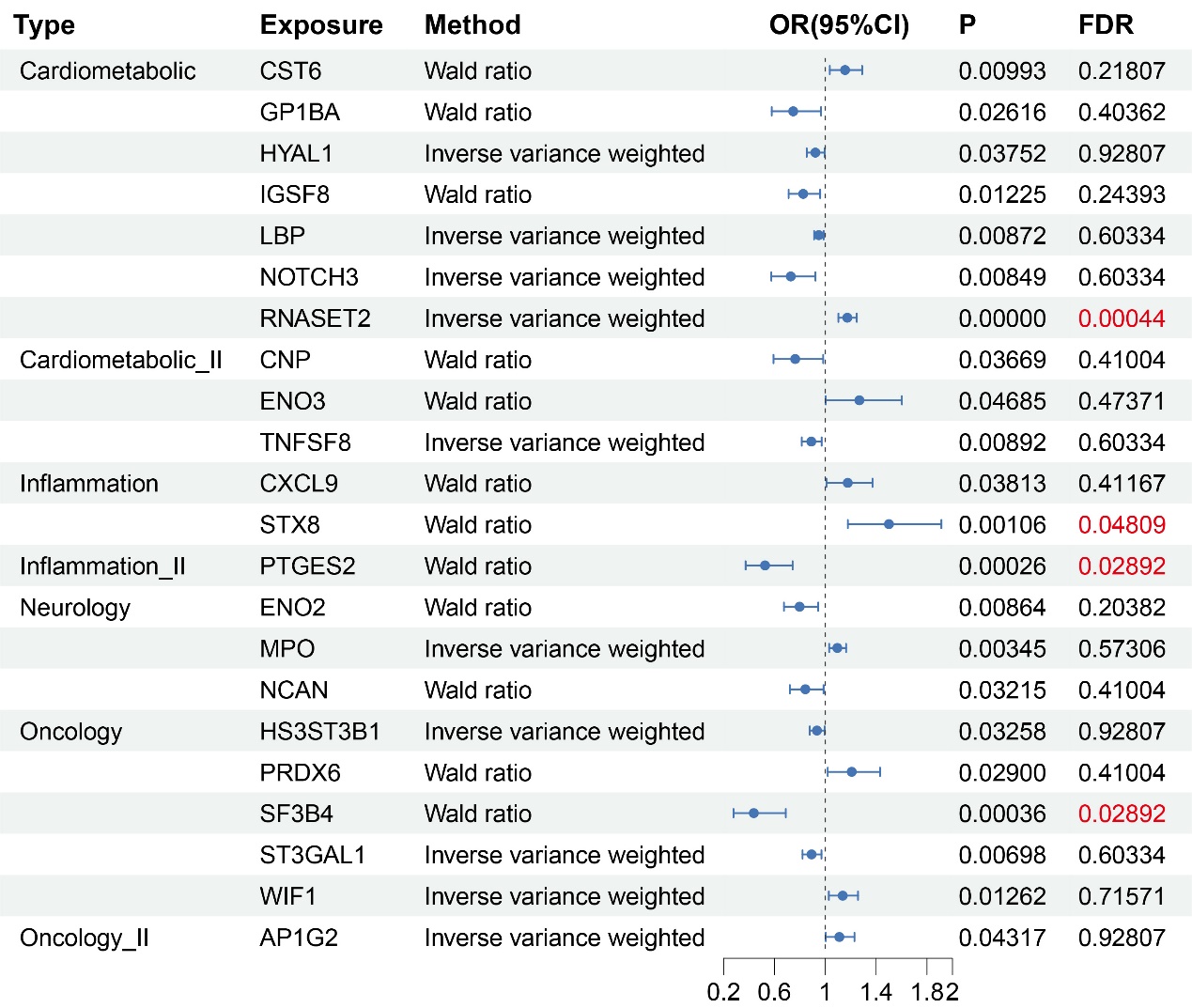


**Figure S1.** Odds ratios (ORs), 95% confidence intervals (CIs), P values, and false discovery rate (FDR) for the effect of plasma proteins on skin cancer estimated using the IVW or Wald ratio approaches of MR.


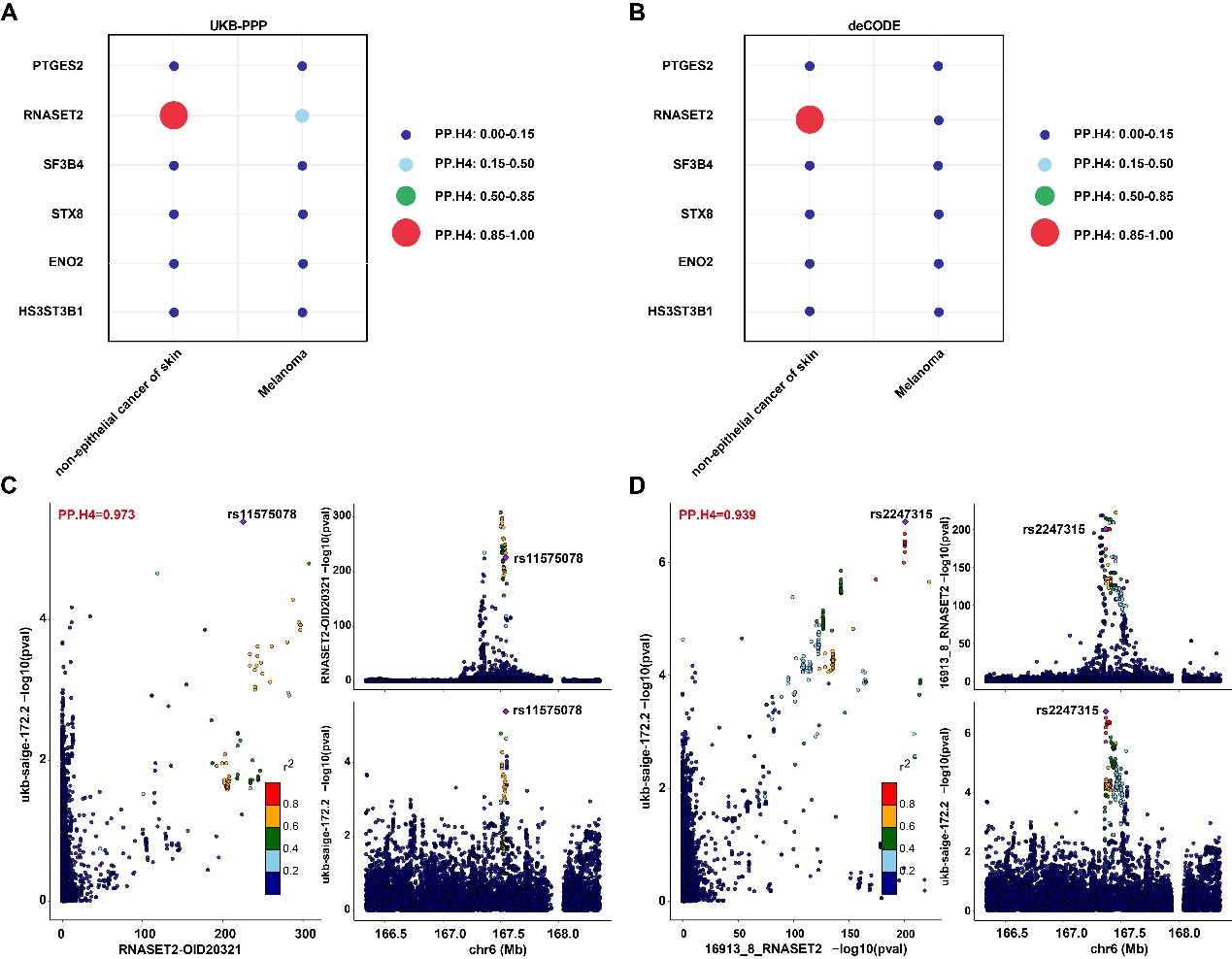


**Figure S2. Colocalization analysis on the associations between plasma proteins and skin cancer subtypes.** (A–B). PH4 values of colocalization analysis for PTGES2, RNASET2, SF3B4, STX8, ENO2, and HS3ST3B1 in the UKB-PPP (A) or deCODE (B) studies with skin cancer subtypes in the Lee Lab of UKB. (C–D). LocusCompare plot for colocalization of pQTL (RNASET2) and non-epithelial skin cancer from the Lee Lab in the UKB-PPP (C) and deCODE (D) studies.


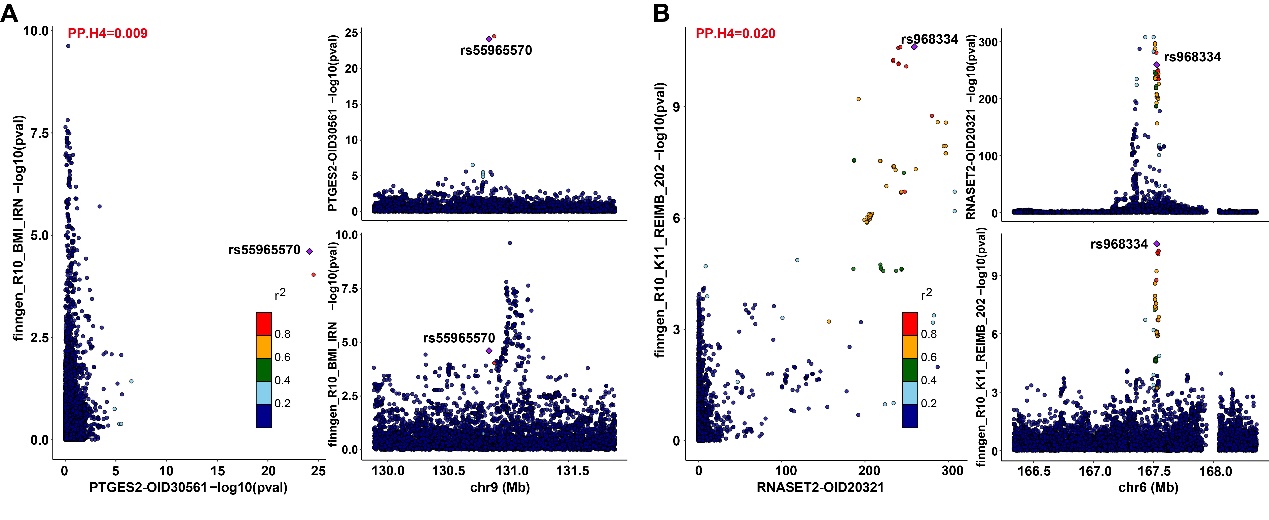


**Figure S3. Colocalization analysis for PTGES2 and BMI or RNASET2 and gastrointestinal endpoints**

**
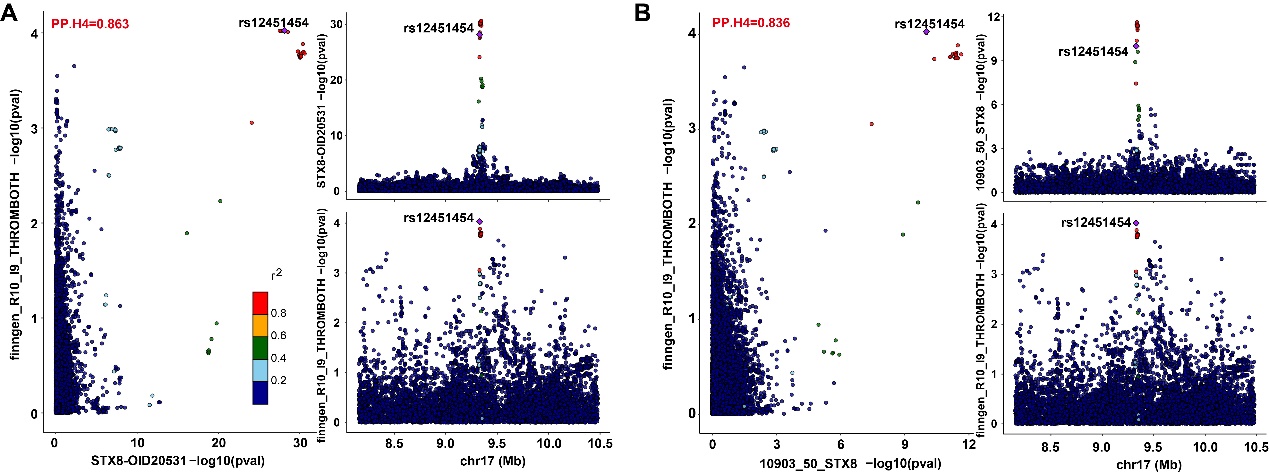
**

**Figure S4. Colocalization analysis for STX8 and other embolism and thrombosis**
